# Untargeted Metabolomics Analysis of the Orchid Species *Oncidium sotoanum* Reveals the Presence of Rare Bioactive C-Diglycosylated Chrysin Derivatives

**DOI:** 10.3390/plants12030655

**Published:** 2023-02-02

**Authors:** Gianluca Zorzi, Sofia Gambini, Stefano Negri, Flavia Guzzo, Mauro Commisso

**Affiliations:** 1 Department of Biotechnology, University of Verona, 37134 Verona, Italy; 2 National Biodiversity Future Center (NBFC), 90133 Palermo, Italy

**Keywords:** chrysin, flavones, flavonoids, untargeted metabolomics, antioxidant activity, MAO-B, *Oncidium sotoanum*, *Orchidaceae*, underexplored species, phytochemicals

## Abstract

Plants are valuable sources of secondary metabolites with pharmaceutical properties, but only a small proportion of plant life has been actively exploited for medicinal purposes to date. Underexplored plant species are therefore likely to contain novel bioactive compounds. In this study, we investigated the content of secondary metabolites in the flowers, leaves and pseudobulbs of the orchid *Oncidium sotoanum* using an untargeted metabolomics approach. We observed the strong accumulation of C-diglycosylated chrysin derivatives, which are rarely found in nature. Further characterization revealed evidence of antioxidant activity (FRAP and DPPH assays) and potential activity against neurodegenerative disorders (MAO-B inhibition assay) depending on the specific molecular structure of the metabolites. Natural product bioprospecting in underexplored plant species based on untargeted metabolomics can therefore help to identify novel chemical structures with diverse pharmaceutical properties.

## 1. Introduction

The search for phytochemicals with pharmaceutical potential is a key aspect of modern medicine, which continuously seeks new therapeutic agents with minimal adverse effects [1]. This research is urgently required due to the emergence of many new diseases, the re-emergence of old ones, and the declining effectiveness of conventional drugs. For example, the overuse and misuse of antibiotics has increased the incidence of microbial infections [2,3] and the prevalence of drug-resistant pathogens [4,5]. Moreover, cancer cells can adapt to anticancer agents, making tumors extremely difficult to eradicate while increasing the risk of toxicity to normal cells caused by chemotherapy [6]. Researchers have therefore turned back to nature in order to expand the search for novel drug candidates [4,7].

Plants offer a large reservoir of bioactive compounds with therapeutic properties. Natural products are structurally diverse and can be classed as alkaloids, phenolics, tannins, glucosinolates, saponins, steroids, terpenes and carotenoids, among others. The natural distribution of such compounds is not equal; phenolic compounds representing 45% of all phytochemicals discovered thus far, followed by terpenoids and steroids (27%) and alkaloids (18%) [8,9]. Plants are thought to produce more than 200,000 different compounds, and even this large number may be an underestimation [10]. Bioprospecting in underexplored plant species may therefore lead to the discovery of novel phytochemicals with desirable pharmaceutical properties.

Flavonoids are phenolic compounds that form a subclass of phenylpropanoids characterized by a common skeleton composed of three rings (C6-C3-C6). These rings can be enzymatically modified to generate chalcones, flavanones, flavones, isoflavones, flavonols, anthocyanidins and flavanols [11]. Flavonoids confer many beneficial effects, including cardiovascular protection, anti-atherosclerosis, anti-inflammation, anticancer and anti-aging activities [12,13,14]. The biological activity of flavonoids has been attributed mainly to their intrinsic antioxidant capacity; this allows them to scavenge free radicals and thus reduce or prevent damage to cells [11]. The specific bioactivity and its potency depend on the decoration of the basic scaffold, mainly with hydroxyl, methoxyl, glycosyl and acyl groups.

Chrysin (5,7-dihydroxyflavone) is a flavone whose bioactivity profile includes antitumor properties [15]. It is particularly active against skin, breast, lung, liver, colon, prostate and pancreatic cancer cells in vitro [1,16]. Chrysin also protects the liver [17,18], attenuates psoriasis-like skin lesions [19], and protects the eye from diseases leading to blindness [20,21] in murine disease models. Molecular docking experiments, sometime supported by in vitro data, suggest that chrysin may be active against influenza virus strain H1N1 [22], enterovirus 71 (EV71) [23], and SARS-CoV-2 [5]. It was also shown to prevent coxsackievirus B3 (CVB3)-induced acute pancreatitis in murine models [24] and inhibit the growth of pathogenic bacteria in vitro [25]. A recent review suggested that, based on in vitro and in vivo data, chrysin may also be neuroprotective [26].

*Oncidium sotoanum* is an ornamental species that produces purple, slightly scented flowers and belongs to the Orchidiaceae family, which is one of the largest families among flowering plants [27]. Historically, the term *sotoanum* was used as a synonym of *ornithorhynchum*, but recent phylogenetic analysis of different *Oncidium* accessions demonstrated that *sotoanum* and *ornithorhynchum* are two different species probably originating in two different zones in the American continent [28]. Although *Oncidium* hybrids are the second most popular varieties of orchids [29], the secondary metabolome of these plants is still poor elucidated, especially for *O. sotoanum*. In *O. baueri*, two recent studies showed that flowers, leaves, pseudobulbs, roots and rhizomes accumulated flavonoid derivatives that belong to the classes of flavanones, such as oncibauerin A and B, and flavones, such as acacetin 7-O-rutinoside and pectolinarigenin-7-O-rutinoside [30,31]. In *O.* “Gower Ramsey” flowers, the red color was ascribed to the presence of anthocyanins based on cyanidin and peonidin aglycones [27].

In this study, we explored the chemical composition of the underexplored orchid species *Oncidium sotoanum* by using an untargeted metabolomics approach to survey the natural compounds produced in various organs. Results revealed high levels of C-diglycosylated chrysin derivatives, which are particularly rare in nature. The metabolic profiling of orchid species could provide a series of leads for the development of new, pharmacologically active natural products.

## 2. Results

### 2.1. Structural Analysis of the Most Abundant O. sotoanum Metabolites by LC-HRMS

**Figure 1:**
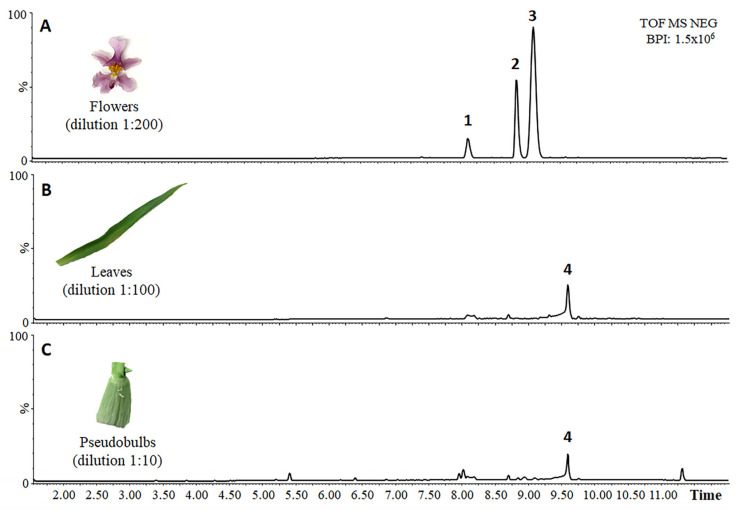
Negative base peak ion chromatograms of diluted *O. sotoanum* flower, leaf and pseudobulb extracts. (**A**) Base peak chromatogram of a flower extract diluted 1:200 (*v*/*v*) and 0.5 µL injected to the LC-HRMS. Peaks numbers stand for a putative chrysin C-(acetyldeoxyhexosyl) hexoside (peak 1) and two putative isoforms of chrysin C-(diacetyldeoxyhexosyl) hexoside (peak 2 and 3). (**B**) Base peak chromatogram of a leaf extract diluted 1:100 (*v*/*v*) and 1 µL injected to the LC-HRMS. Peak 4 indicates a putative chrysin C-(triacetyldeoxyhexosyl) hexoside-O-hexoside. (**C**) Base peak chromatogram of a pseudobulb extract diluted 1:10 (*v*/*v*) and 1 µL injected to the LC-HRMS.

We prepared methanol extracts of *O. sotoanum* flowers, leaves and pseudobulbs for LC-HRMS analysis (UPLC-PDA-ESI/QqTOF) following an untargeted metabolomics approach. The resulting chromatograms are shown in Figure 1.

Flowers (Figure 1A) accumulated three major compounds, the first with an *m*/*z* value of 603.1723 and a retention time of 8.08 min (peak 1), and the other two with the same *m*/*z* value of 645.1820 and retention times of 8.82 min (peak 2) and 9.06 min (peak 3). Leaves and pseudobulbs (Figure 1B,C) accumulated one major compound with an *m*/*z* value of 849.2453 and a retention time of 9.55 min (peak 4). The absorption spectra of these metabolites showed a major peak at 267 nm and a less intense one at 313 nm, which are characteristic of flavones [32]. The metabolites were identified by inspecting their fragmentation patterns as determined using the FAST-DDA function available in MassLynx.

**Figure 2:**
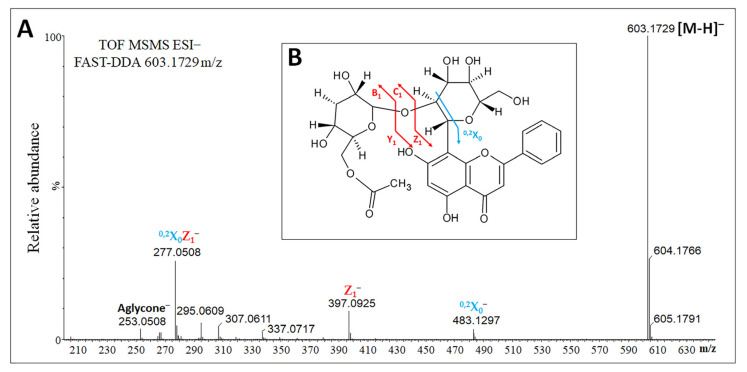
Fragmentation profile and identity of the ion with an *m*/*z* value of 603.1729. (**A**) Fragmentation profile determined by FAST-DDA. (**B**) Putative structural annotation as chrysin C-(acetyldeoxyhexosyl) hexoside. Cyan and red arrows indicate the breakage points, giving rise to the diagnostic fragments highlighted by the glycoside fragmentation nomenclature [33].

Compound 1 (Figure 2A) produced a base peak ion of *m*/*z* = 483.1291 (^0,2^X_0_^−^) by the loss of 120 Da (suggesting the cross-ring cleavage of a hexose) and an ion of *m*/*z* = 397.0932 (Z_1_^−^) by the loss of 206 Da, which matches an acetyl hydrated deoxyhexose (42 Da + 18 Da + 146 Da) moiety (Figure 2B). The daughter ion (*m*/*z* = 397.0932) may therefore represent a dehydrated C-glycosylated flavone, as reported for the fragmentation of vitexin-2″-*O*-rhamnoside [33]. Neutral losses of 60, 90 and 120 Da were observed for an ion of *m*/*z* = 397.0932, resulting in the production of three fragments of *m*/*z* = 337.0688 [M-206-60]^−^, 307.0594 [M-206-90]^−^ and 277.0504 (^0,2^X_0_Z_1_^−^) [M-206-120]^−^, with the last being the most abundant. These neutral losses are characteristic of glycosides linked via carbon–carbon (CC) bonds. A fragment of *m*/*z* = 253.0505 was also detected, differing by 24 Da from the ion with an *m*/*z* value of 277.0504. Previously, the main fragment of vitexin-2′′-*O*-rhamnoside (*m/z* = 577) was shown to have an *m*/*z* value of 293, consisting of the aglycone apigenin (*m*/*z* = 269) plus 24 Da [33]. These data suggest that compound 1 is an aglycone (254 Da) C-glycosylated with a hexose, which is in turn attached to a deoxyhexose, and is probably acetylated at one hydroxyl group of the sugar moiety.

**Figure 3:**
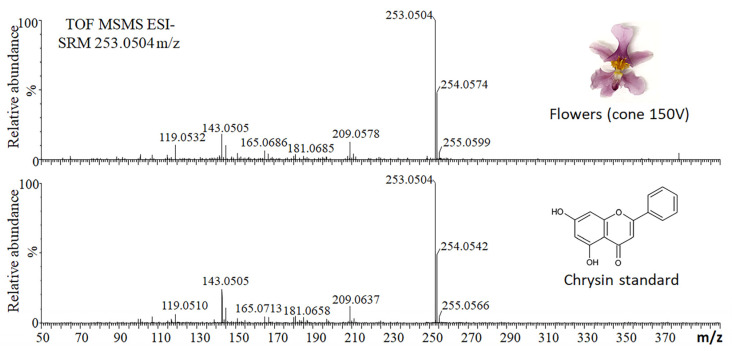
Selected reaction monitoring (SRM) profile of the ion with an *m*/*z* value of 253.0504. The flower extract was analyzed in negative ionization mode at a high cone voltage (150 V) to induce the in-source fragmentation of larger metabolites and release the aglycone. The same analysis was applied to the chrysin standard (1 ng/µL). Collision induced dissociation (CID) was carried out using argon gas at 35 eV.

The aglycone of compound 1 was characterized by selective fragmentation of the *m/z* = 253 ion [aglycone-H]^−^, which was achieved using the selected reaction monitoring (SRM) method and a high cone voltage (150 V) to induce in-source fragmentation. The results showed a peculiar fragmentation profile that was highly similar to that of a chrysin authentic commercial standard (Figure 3). Therefore, compound 1 was annotated as chrysin C-(acetyldeoxyhexosyl) hexoside (Figure 2B).

**Figure 4:**
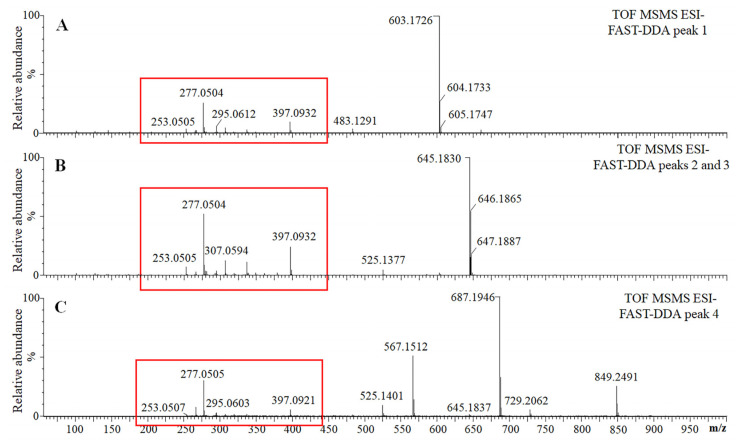
FAST-DDA analysis of four unidentified compounds. The analysis of compounds 1 (**A**), 2 and 3 (**B**), and 4 (**C**) showed that compounds 2 and 3 share the same *m*/*z* value (645.1820) but eluted at 8.82 and 9.06 min, respectively. Red boxes indicate the same core structure among the four compounds.

Compounds 2 and 3 shared the same *m*/*z* value (645.1820) at different retention times and the same fragmentation profiles, suggesting that they might be structural isomers. The collision-induced dissociation (CID) of both ions (*m*/*z* = 645.1820 [M-H]^−^) produced fragments of *m*/*z* = 603.1726 [M-42-H]^−^ and 525.1377 [M-120-H]^−^, suggesting the presence of an acetyl group and a C-glycoside. The detection of an ion with an *m*/*z* value of 397.0932 indicated a neutral loss of 248 Da, suggesting the presence of a hydrated deoxyhexose joined to two acetyl groups (164 Da + 42 Da + 42 Da). Other daughter ions, such as those of *m*/*z* = 337.0726, 307.0594, 277.0504, 295.0612 and 253.0505, indicated the presence of the same core structure detected in compound 1 (Figure 4A,B). These data strongly suggest that compounds 2 and 3 are structural isomers of chrysin C-(diacetyldeoxyhexosyl) hexoside.

Compound 4 had an *m*/*z* value of 849.2487 and produced a major fragment ion of *m*/*z* = 687.1964, revealing a neutral loss of 162 Da compatible with the release of an *O*-hexose. This glycosyl residue is probably esterified with a hydroxyl group at position 5 or 7 of the aglycone A ring. Moreover, the fragment ion of *m*/*z* = 645.1837 differs by 42 Da from 687.1964, suggesting the presence of an acetyl group. The other two visible fragments of *m*/*z* = 567.1508 and 525.140 represented losses of 120 Da, suggesting a cross-ring cleavage of hexose from ions with *m*/*z* values of 687.1964 and 645.1837, respectively. Similar to compounds 2 and 3, compound 4 generated the same core fragments at lower *m*/*z* values, suggesting a common basic structure (Figure 4C). Therefore, compound 4 was identified as chrysin C-(triacetyldeoxyhexosyl) hexoside-*O*-hexoside.

### 2.2. Untargeted Metabolomics Reveals the Existence of Other Chrysin Derivatives

The LC-MS data files for the flower, leaf and pseudobulb samples were processed using Progenesis QI, resulting in a final data matrix with 457 rt/mz features and 61 putatively identified metabolites (Supplementary File S1). Most of the identified metabolites were flavones, with apigenin and chrysin as the two most representative aglycones. Chrysin derivatives were putatively identified by comparing their absorbance peaks and fragments with those of the chrysin authentic commercial standard. We induced in-source fragmentation as described above with the specific aim of fragmenting the aglycone (*m*/*z* = 253.0505) in negative ionization mode. This revealed 26 putative chrysin derivatives in leaves and pseudobulbs (Table 1).

**Figure 5:**
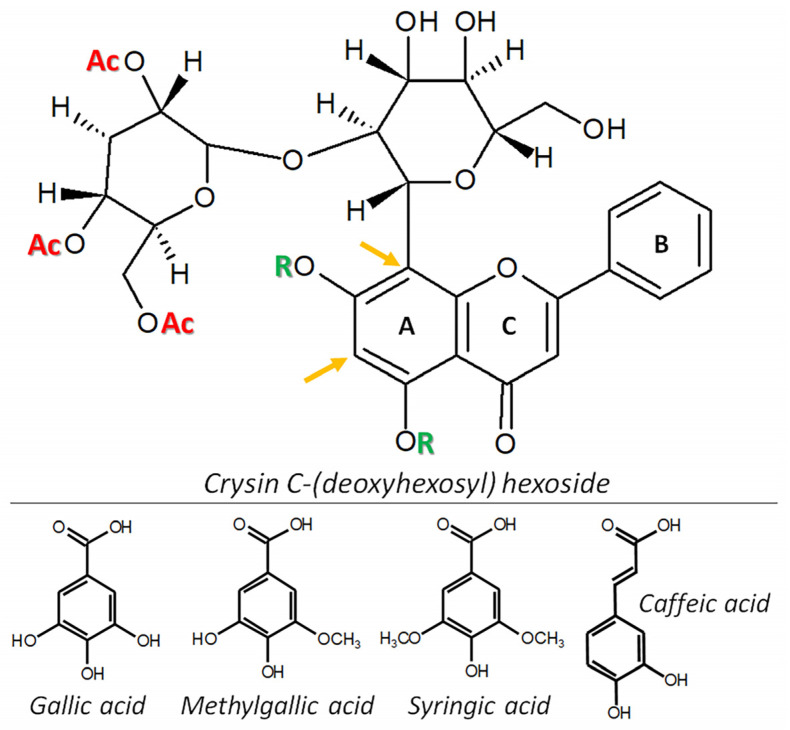
Proposed core structure for chrysin derivatives in *Oncidium sotoanum*. The chrysin flavone is linked to a hexose via a CC bond. This sugar residue is, in turn, attached to a deoxyhexose. Ac = potential sites of acetylation and acylation with syringic or gallic acid. The orange arrows highlight possible positions of the C-bond between the sugar residue and chrysin (6-C or 8-C of the flavone A ring). R = possible esterification sites with a sugar that can be acylated with caffeic, gallic and methylgallic acid.

Most of these metabolites shared the main crysin C-(deoxyhexosyl) hexoside core structure, but the deoxyhexose moiety was decorated with a variety of acetyl and/or acyl groups, such as syringic or gallic acid. In some of the chrysins, a glycoside was esterified to one of the two hydroxyl groups of the flavone structure. In some cases, the *O*-glycoside was modified further with caffeic, gallic or methylgallic acid. The various combinations are shown in Figure 5.

### 2.3. Chrysin Quantification

Absolute quantification of chrysin derivatives in flowers, leaves and pseudobulbs was carried out by comparing the absorption at 267 ± 4 nm with a calibration curve of the chrysin authentic commercial standard. The precise levels are expressed as chrysin equivalents in Table 2, representing the sum of the amounts of peaks 1, 2, 3 and 4 from Figure 1. The other chrysin derivatives detected by LC-MS were not taken into account because the photodiode array (PDA) signal was too low. An *O. sotoanum* flower was found to weigh 53.2 ± 2.6 mg on average, so 19 flowers were needed to prepare 1 g of fresh weight (FW).

### 2.4. Antioxidant and MAO-B Inhibition Assays

**Figure 6:**
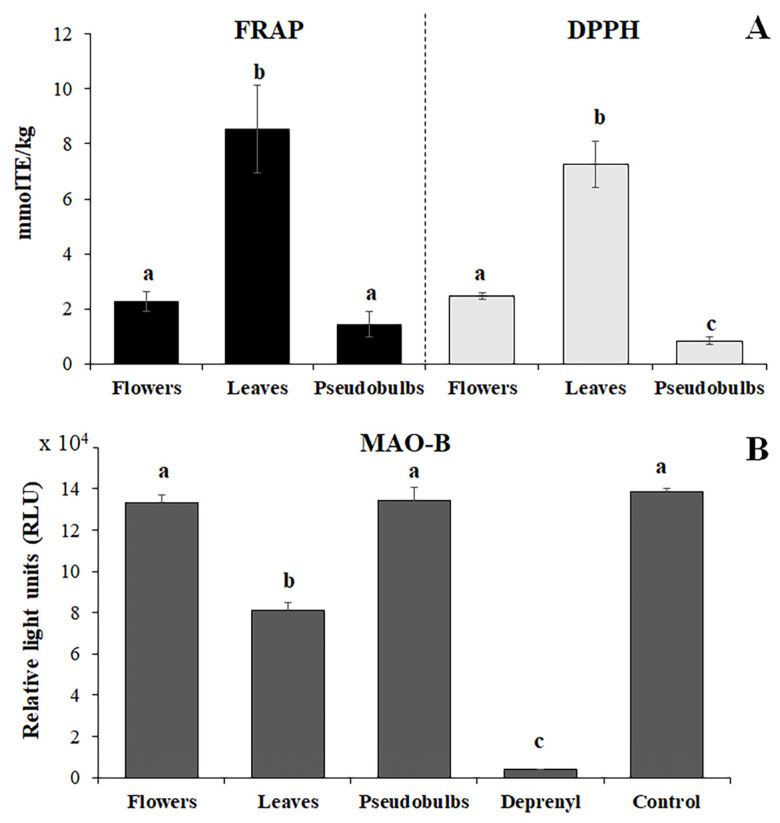
Antioxidant and MAO-B inhibition assays of flower, leaf and pseudobulb extracts. (**A**) Antioxidant (FRAP, DPPH) assays, with values expressed as Trolox equivalents. (**B**) MAO-B assay using undiluted samples, with values expressed as relative light units (RLUs). In this assay, 2.5 μM deprenyl was used as a positive control, and MAO buffer without inhibitor as a negative control. Data are means ± standard deviation (*n* = 3, one-way ANOVA with Tukey’s post hoc test, and different lowercase letters represent statistically significant differences between groups, *p* < 0.05).

The antioxidant activity of flowers, leaves and pseudobulbs was evaluated using FRAP and DPPH assays (Figure 6). The antioxidant capacity of leaf extracts was higher than that of pseudobulbs or flowers in both assays. However, even though the antioxidant capacity of flowers was higher in the DPPH assay than the FRAP assay, the reverse was true for the other two organs.

Given that chrysin has been reported as a neuroprotective agent [26], we carried out an in vitro monoamine oxidase (MAO-B) inhibition assay on flower, leaf and pseudobulb extracts using deprenyl as a positive control and buffer without inhibitor as a negative control. We found that non-diluted leaf extracts significantly inhibited MAO-B whereas the other extracts had a negligible effect (Figure 6B). Chrysin derivatives in flowers and pseudobulbs therefore appear to lack MAO-B inhibition activity. Moreover, serial dilutions of leaf extracts (1:5 and 1:10 *v*/*v* in MAO buffer) revealed no inhibition even at the lowest dilution factor, suggesting leaf metabolites possess only weak MAO-B inhibition activity (data not shown).

**Figure 7:**
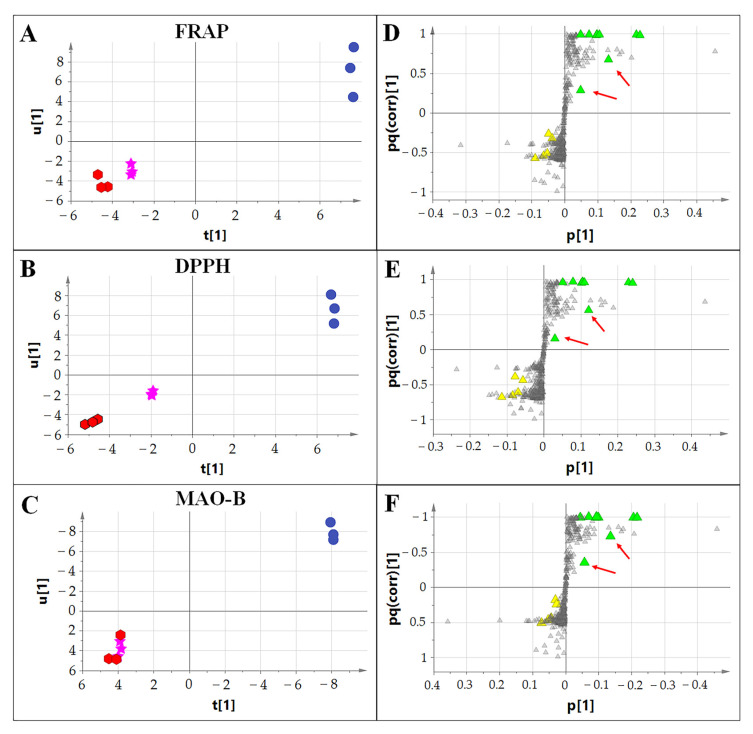
OPLS analysis showing correlation between the metabolites, antioxidant capacity and MAO-B activity of different extracts. (**A**–**C**) are the score scatter plots of the FRAP, DPPH and MAO-B assays, respectively. Red hexagons indicate pseudobulb samples, pink stars the flowers and blue circles the leaves. (**D**–**F**) are the loading plots of the FRAP, DPPH and MAO-B assays, respectively. Triangles indicate the metabolites. Yellow triangles are chrysin derivatives in which the acyl group (syringic or gallic acid) is linked to the deoxyhexose of the C-diglycoside (Ac in Figure 5). Green triangles indicate those chrysin derivatives with gallic, methylgallic or caffeic acid attached to the *O*-glycoside (R in Figure 5). The red arrows indicate the two chrysins including methylgalloyl residues attached to the *O*-glycoside.

Finally, orthogonal partial least squares (OPLS) multivariate statistical analysis was used to investigate the correlation between metabolite levels, antioxidant activity and MAO-B inhibition capacity in the extracts. The peak values in the data matrix were converted to percentages after setting the total ion signal of each sample to 100% to account for the different extraction volumes, dilutions and injection volumes used for each tissue. The score scatter plot showing t[1] vs. u[1] highlighted a linear correlation between the metabolites (X variables) and the antioxidant activity (Y variable) determined using the FRAP assay (Figure 7A) and DPPH assay (Figure 7B). The corresponding loading plots (Figure 7D,E), which showed the p[1] vs. pq(corr)[1], revealed specific metabolites correlating with antioxidant activity.

The results showed that only the chrysins with gallic or caffeic acid attached to the sugar moiety linked via an *O*-glycosidic bond to the A ring of the flavone aglycone were strongly correlated with antioxidant activity. Such structures were abundant in the leaves but not in the flowers, despite the generally high levels of chrysins in flowers. These data confirm that chrysin bioactivity is dependent on the precise decoration of the basic chrysin scaffold. In leaves, chrysins often feature a galloyl residue attached to the *O*-glycoside, whereas the further methylation of one hydroxyl group in the galloyl residue seems to reduce the antioxidant capacity (Figure 7D,E). Similarly, the OPLS score scatter plot showed a difference between extracts in terms of MAO-B inhibition (Figure 7C), and the loading plot suggested, even in this case, that chrysins with galloyl residues attached to the *O*-glycoside might show a greater degree of MAO-B inhibition activity (Figure 7F).

## 3. Discussion

Untargeted metabolomics analysis of *O. sotoanum* flower, leaf and pseudobulb extracts revealed 26 different chrysin derivatives, all based on a unique common structure of chrysin C-(deoxyhexosyl) hexoside. We tentatively annotated the four most abundant molecules as chrysin C-(acetyldeoxyhexosyl) hexoside, two structural isomers of chrysin C-(diacetyldeoxyhexosyl) hexoside and chrysin C-(triacetyldeoxyhexosyl) hexoside-*O*-hexoside. These structures are rare in nature, and only a few species produce similar chrysins to those found in *O. sotoanum*. One example was identified in the leaves of *Sarcotheca griffithii* (family Oxalidaceae) by LC-MS analysis, which revealed an *m*/*z* value of 563.17611 [M+H]^+^ and a fragmentation pattern similar to the chrysin C-(*O*-deoxyhexosyl) hexoside found in our extracts. Additional ^1^H NMR analysis [34] confirmed the identity of chrysin 6-C-(2″-*O*-α-l-rhamnosyl)-β-glucoside. Other chrysins with similar structures include chrysin-8-C-(2″-*O*-α-6- deoxyglucosyl)-β-glucoside found in the peel tissue of *Passiflora edulis* Sim fruits [35], chrysin-6-arabinosyl-8-C-glucoside and chrysin-7-*O*-glucuronide found in *Scutellaria immaculata* and *S. ramosissima* aerial parts and root tissue [36], chrysin-7-*O*-β-glucoside found in *Cytisus villosus* Pourr [37] and chrysin 6-C-β-glucosyl-8-C-β-glucuronoside, chrysin-7-*O*-gentiobioside and chrysin-6-C-β-glucosyl-8-C-α-arabinoside found in *Oroxylum indicum* seeds [38]. Acetylated forms of chrysin, such as chrysin 7-(6″-*O*-acetyl)-*O*-β-glucoside and chrysin 7-(4″-*O*-acetyl)-*O*-β-glucoside, were identified in *Calicotome villosa* by NMR spectroscopy [39].

The two major sources of chrysin in nature are honey and propolis. The chrysin content of honey ranges from 0.22 µg/g in Wolfberry honey from China [1] to 1.2 µg/g (chrysin plus galangin) in *Rubus* honey [40], 1.31 µg/g in Manuka Honey [1] and 1.53 µg/g in Forest honey from Spain [41]. Chrysin is also found in 10 different buckwheat honeys at concentrations of 0.19–1.28 µg/g [42]. Much higher levels are found in propolis, reaching 10 mg/g in Greek propolis, 11 mg/g in Korean propolis, 19 mg/g in Polish propolis, 66.3 mg/g in Bulgarian propolis and 82.9 mg/g in Hungarian propolis [43,44,45,46]. Other minor sources of chrysin are listed in Table 3.

The presence of chrysin in propolis and honey strongly suggests that many plant species accumulate chrysins in flowers, probably in the pollen and/or nectar [54,55]. Accordingly, we found that *O. sotoanum* accumulates high levels of glycosylated chrysins in flowers, reaching bulk levels of 10 mg/g FW. However, honey and propolis include chrysin aglycones that are probably derived from glycosylated forms by the enzymatic activity of honeybee saliva on flavonoids [56]. The high chrysin content of flowers supports the hypothesis that crysins might be involved in reproduction in *O. sotoanum* and perhaps in other plant species.

Flavonoids are phenolic compounds that confer positive health effects in humans due to their antioxidant capacity. Chrysin aglycone is a strong antioxidant based on ABTS and DPPH assay data [57,58], reflecting its ability to scavenge superoxide radicals [59]. However, chrysin has a lower antioxidant capacity than other flavones such as luteolin, which has been attributed to the lack of hydroxyl groups in the B ring [60]. We assessed the antioxidant capacity of *O. sotoanum* flower, leaf and pseudobulb extracts, which accumulated different sets of metabolites. Leaf extracts showed the greatest antioxidant activity in FRAP and DPPH assays. OPLS analysis revealed that only chrysins featuring hydroxybenzoic or hydroxycinnamic acid linked to the *O*-glycosyl moiety were strong antioxidants. These structures were abundant in leaves, which correlated well with the FRAP and DPPH assay data. Accordingly, the common structure of chrysin C-(deoxyhexosyl) hexoside does not appear to contribute much to the antioxidant capacity of the extracts. Indeed, C-glycosylated flavonoids possess lower antioxidant activity than *O*-glycosylated forms, especially those conjugated in the A-ring [61,62]. Furthermore, the presence of hydroxybenzoyl and hydroxycinnamoyl residues (especially gallic acid) attached to the *O*-glycoside strongly enhanced the antioxidant activity compared to non-acylated counterparts, as previously reported [63,64,65]. Interestingly, the type of linkage between chrysin and the sugar residues seems to strongly affect the FRAP and DPPH results, regardless of the type of attached hydroxybenzoic acid. In fact, galloyl moieties joined to the C-diglycoside showed less antioxidant activity than the same moieties linked to the *O*-glycoside, which should be explored in more detail in future investigations.

Chrysin may also confer neuroprotection, slowing the pace of cognitive decline in neurodegenerative disorders such as Alzheimer’s disease and Parkinson’s disease [26]; the latter has been demonstrated in vivo using transgenic *Caenorhabditis elegans* disease models [66]. The administration of 5 and 20 mg/kg chrysin every 28 days also increased sucrose consumption and reduced immobility during the tail suspension test in female C57B/6J mice exposed to chronic unpredictable mild stress as a model of depression [67,68,69]. Other in vivo investigations in rodent depression models indicated antidepressant-like effects following the administration of chrysin [70]. Neurodegenerative disease and depression are both associated with the loss of MAO activity [71]. MAOs oxidize endogenous and exogenous monoamine neurotransmitters, producing reactive oxygen species and triggering oxidative stress. Humans produce two isoforms of MAO known as MAO-A and MAO-B, with the latter active in the brain [72]. Selective MAO-B inhibitors can therefore slow the progression of neurodegenerative disorders, and are also effective for atypical or treatment-resistant depression [73]. Chrysin has been shown to inhibit MAO-B in a concentration-dependent manner with IC_50_ values of 12.3 μM [74], 0.79 μM [75] and 1.04 μM [76]. We did not evaluate the IC_50_ of *O. sotoanum* extracts due to the poor MAO-B inhibition responses that occurred, even with undiluted samples. This result appears to contradict previous findings. However, this discrepancy may reflect the fact that previous experiments tested only chrysin aglycone, whereas all our chrysins were glycosylated and acylated. *O. sotoanum* flowers contain mainly C-diglycosylated and acylated chrysins, and MAO-B inhibition by flower extracts was negligible. The decoration of the chrysin structure therefore appears to have a profound effect on MAO-B inhibition. Finally, the presence of gallic acid attached to the *O*-glycosyl moiety appeared to favor MAO-B inhibition, and this should be investigated in more detail.

The potential health benefits of chrysin depend strongly on its pharmacokinetic and pharmacodynamic profiles in the human body. The administration to human volunteers of 400 mg chrysin in a single dose resulted in extensive plasma binding (>99%) and oral bioavailability of 0.003–0.02% [77]. After absorption, chrysin is extensively converted by phase II metabolism into chrysin 7-*O*-sulfate and chrysin 7-*O*-glucuronide, as observed in studies using human intestinal Caco-2 cells, male Sprague-Dawley rats and human volunteers. Moreover, these forms are mainly excreted in the feces [77,78,79], suggesting that chrysin is poorly absorbed and rapidly metabolized and eliminated, resulting in low bioavailability [77]. In addition, chrysin is poorly soluble in water [80], which limits its applications in healthy food [81] and makes it more difficult to achieve the recommended daily amount of 0.5–3.0 g [82]. Furthermore, even low amounts of chrysin were found to be toxic in a liver cell line [82,83], probably due to the activity of peroxidase-like enzymes that generate toxic chrysin derivatives [83]. Therefore, further studies on the toxic effects of chrysin are required. However, many of the previously cited pharmacological studies refer to chrysin aglycone, whereas no data are available for C-diglycosylated chrysins. C-glycosylated flavonoids are resistant to hydrolysis, and no mammalian enzymes that cleave the C-glycosidic linkage have been discovered thus far. C-glycosylated flavonoids might therefore act as probiotics, serving as substrates for bacteria resident in the human colon [84]. Interestingly, C-multiglycosylated flavonoids are rapidly and easily absorbed in the intestine, unlike the C-monoglycosylated forms, and they can be distributed to other tissues where they may exert pharmacological effects [61]. Because *O. sotoanum* chrysins are mainly C-diglycosylated forms, they could be absorbed and distributed in the body, bypassing the problem of low bioavailability and conferring the abovementioned health benefits. However, the hydrolysis of the *O*-glycoside group, the part responsible for antioxidant and MAO-B inhibition activity, might reduce the bioactivity of these metabolites.

## 4. Materials and Methods

### 4.1. Sample Collection and Preparation

Six *O. sotoanum* plants were purchased from an Italian orchid nursery and cultivated in phytotrons at 25 ± 2 °C and 70% relative humidity with a 16 h photoperiod. Flowers, leaves and pseudobulbs were collected 10 days after anthesis; nine flowers, six pseudobulbs and six leaves were used to create each sample, defined as a biological replicate. Almost 30 flowers were weighed on an analytical balance. The samples were immediately frozen in liquid nitrogen, ground with a mortar and pestle, and 100 mg of frozen powder was extracted with six volumes (*w*/*v*) of 100% LC-MS grade methanol (Honeywell, Seelze, Germany). The samples were vortexed for 30 s, sonicated for 10 min in a 40-kHz ultrasonic bath (SOLTEC, Milano, Italy) with ice, and centrifuged at 14,000× *g* for 15 min at 4 °C. The supernatants were stored at −20 °C.

### 4.2. Untargeted Metabolomics Analysis

Methanol extracts of flower, leaf and pseudobulb samples were diluted to 1:200, 1:100 and 1:10 with LC-MS grade water (Honeywell) and then passed through 0.22-μm Minisart filters (Sartorius-Stedim Biotech, Göttingen, Germany). LC-HRMS analysis was carried out as previously described [85]. Briefly, an ACQUITY I CLASS UPLC system with an ACQUITY PDA detector was connected to an electrospray ionization (ESI) source and Xevo G2-XS qTOF mass spectrometer (all equipment from Waters, Manchester, UK). The PDA comprised an eλ detector (190–800 nm) and a sensitive flow cell. The samples were fractionated in a Waters reversed-phase BEH C18 column (2.1 mm × 100 mm, 1.7 µm) with a Vanguard column (2.1 mm × 5 mm, 1.7 µm) at 30 °C. We used a gradient of solvent A, which was 0.1% formic acid (Biosolve Chimie, Dieuze, France) in LC-MS grade water, and solvent B, which was 100% acetonitrile, at a flow rate of 0.350 mL/min. The gradient started with 1% B, held to 1% B for 1 min, then increased to 40% B at 10 min, to 70% B at 13.5 min, and to 99% at 14 min. Subsequently, the method remained in 99% B for 2 min and was then decreased to 1% B at 16.1 min. The method remained in isocratic (1% B) for 3.9 min and ended at 20 min. Samples were placed in an ACQUITY flow through needle autosampler kept at 8 °C. We injected 0.5 µL of each flower sample and 1 µL of the other samples. The ESI source parameters were as previously described [86] and samples were ionized in negative mode. Positive ionization was also induced to facilitate identification. MS data were acquired in continuum and sensitivity modes. The scan range was set to 50–2000 *m*/*z* and the scan time at 0.3 s. MS data were acquired using function 1 (no fragmentation) and function 2 (CID with argon gas at a collision energy of 35 eV). Samples were analyzed by FAST-DDA and SRM in negative ion mode. FAST-DDA analysis was carried out by setting the dual-dynamic collision energies to 10–40 eV for low-mass collision energy and 20–80 eV for high-mass collision energy. Automatic switching to MS/MS mode was enabled when the total ion current intensity rose above 100,000/s, switching off after 1 s. A tolerance window of ±3.0 Da and a peak extract window of 2.0 Da were set in deisotope peak detection mode. For SRM analysis, the ESI cone voltage was set at 150 V to induce in-source fragmentation. MS data were acquired using function 1 as described above, whereas function 2 was set to a scan range of 50–400 *m*/*z*, a scan time of 0.1 s, a selected mass of 253 *m*/*z* and a collision energy ramp of 15–60 eV. All functions were controlled using Masslynx v4.1 (Waters). Instrument accuracy was checked by infusing a solution of 100 pg/µL leucine-enkephalin at a flow rate of 10 µL/min and generating a signal of 556.2771 in positive mode and 554.2615 in negative mode. The raw MS data files were processed using Progenesis QI (Waters) to obtain a final data matrix.

### 4.3. Chrysin Quantification

Chrysin was quantified using the peak areas of the four most abundant chrysin derivatives visible in the chromatograms (Figure 1). The PDA chromatograms at 267 ± 4 nm and an automatic integration function available in Masslynx v4.1 were used to determine the peak areas. Chrysin commercial standards (Sigma-Aldrich, St Louis, MO, USA) at 0.001, 0.01, 0.05, 0.1, 0.25, 0.5, 0.75, 1 and 2 ng were injected into the LC-HRMS system twice to create a calibration curve (y = 1486.2x − 71.112) with an r^2^ value of 0.9986. The final amounts were reported as mg/g FW of chrysin equivalents.

### 4.4. DPPH and FRAP Antioxidant Assays

Antioxidant activity was assessed using both FRAP and DPPH in vitro assays, as previously described [87]. Briefly, 20 µL of each crude or diluted methanol extract (Table 4) was tested in triplicate in a 96-well microplate (Sarstedt, Nümbrecht, Germany) by adding 200 µL of test reagent (FRAP solution or 100 µM DPPH in 70% methanol). Absorbance was recorded using an Infinite 200 Pro Microplate reader (Tecan Italia, Cernusco sul Naviglio, Italy) at 593 nm after incubation for 15 min at 37 °C (FRAP) or at 515 nm after incubation for 30 min at 25 °C in the dark (DPPH). For both assays, antioxidant activity was expressed as µmol/100 g FW in comparison to a Trolox (Sigma-Aldrich, St Louis, MO, USA) calibration curve.

### 4.5. MAO-Glo Assay

MAO-B activity was tested using a two-step bioluminescent MAO-Glo assay (Promega, Milan, Italy) as previously reported [88]. Briefly, 250 µL of flower, leaf or pseudobulb methanol extract was placed in a speedvac (Heto-Holten, Frederiksborg, Denmark) to remove the solvent, and the dry pellets were solubilized in 250 µL of MAO reaction buffer, comprising 100 mM HEPES (pH 7.5), 5% (*v*/*v*) glycerol and 10% (*v*/*v*) dimethyl sulfoxide (DMSO). Samples were used undiluted or diluted to 1:5 and 1:10 (*v*/*v*) with the MAO reaction buffer. MAO-B inhibition was tested by adding 12.5 µL of MAO buffer containing 4 µM MAO-B substrate and 12.5 µL of the candidate inhibiting solution in 96-well flat-bottom white opaque plates (Thermo Fisher Scientific, Rodano, Italy). The reaction was initiated by adding 25 µL MAO buffer containing 20 µg/mL of MAO-B. The 50-µL reaction was incubated for 1 h at room temperature, then mixed with 50 µL of luciferin detection reagent and incubated for 20 min at room temperature. Luminescence was recorded on an Infinite 200 Pro microplate reader. Three technical replicates were analyzed for each sample. We used 12.5 µL of the irreversible MAO-B inhibitor l-deprenyl/selegiline (Sigma-Aldrich, St Louis, MO, USA) at a concentration of 2.5 µM in MAO reaction buffer as a positive control and 12.5 µL of the MAO reaction buffer as a negative control. Blank samples were also included in the experiment and contained only the MAO reaction buffer without the enzyme.

### 4.6. Statistical Analysis

Multivariate statistical analysis was applied to the LC-MS data matrix (output from Progenesis QI) using Umetrics SIMCA 13.0 (Sartorius-Stedim Biotech). The data matrix included “n” observations (samples) and X variables (relative abundances of detected metabolites). The FRAP, DPPH and MAO-B inhibition values were added and considered as Y variables. Prior to analysis, the X variables were mean centered and PARETO transformed, whereas the Y variables were mean centered and UV transformed. OPLS analysis was carried out to detect correlations between the X and Y variables (i.e., which metabolites correlated with the antioxidant and MAO-B inhibition activities). The final OPLS model was checked by 400 permutations and CV-ANOVA (*p* < 0.01) tests. The FRAP, DPPH and MAO-B inhibition data were validated by ANOVA followed by a post hoc Tukey’s test (*p* < 0.05).

## 5. Conclusions

In this study, we have demonstrated that *O. sotoanum*, an ornamental orchid species producing purple flowers, mainly accumulates derivatives of chrysin C-(deoxyhexosyl) hexoside, which are rarely found in nature and would act as antioxidants and weak MAO-B inhibitors when acylated at the *O*-glycosyl moiety. Although C-diglycosylated flavonoids should be more easily absorbed than C-monoglycosides, the pharmacokinetic and pharmacodynamic profiles of C-diglycosylated chrysin require further investigation. The possible hydrolysis of the *O*-glycoside moiety in the gut may also reduce the bioactivities of these metabolites after ingestion.

The exploitation of the untargeted metabolomics approach combined with the use of LC-HRMS technique allows to deep investigate the metabolic profiles of underexplored plant species, which may be precious sources of healthy metabolites.

## Figures and Tables

**Table 1:** Chrysin derivatives putatively identified in *Oncidium sotoanum* organs.

r.t.	*m*/*z* Negative	*m*/*z* Positive	Formula	Putative Identification
6.03	577.1569	579.1714	C_27_H_30_O_14_	Chrysin-C-hexoside-C-hexoside
6.82	561.1611	563.1765	C_27_H_30_O_13_	Chrysin-C-(O-deoxyhexosyl) hexoside
7.08	811.2311 *	767.2399	C_35_H_42_O_19_	Chrysin-C-(O-monoacetyldeoxyhexosyl) hexoside-O-hexoside
7.36	917.2367	919.2508	C_42_H_46_O_23_	Chrysin-C-(O-monoacetyldeoxyhexosyl) hexoside-O-galloyl hexoside
7.39	603.1722	605.1870	C_29_H_32_O_14_	Chrysin-C-(O-monoacetyldeoxyhexosyl) hexoside 1
7.51	603.1723	605.1870	C_29_H_32_O_14_	Chrysin-C-(O-monoacetyldeoxyhexosyl) hexoside 2
8.05	921.2301 **	809.2512	C_37_H_44_O_20_	Chrysin-C-(O-diacetyldeoxyhexosyl) hexoside-O-hexoside 1
8.07	603.1723	605.1870	C_29_H_32_O_14_	Chrysin-C-(O-monoacetyldeoxyhexosyl) hexoside 3
8.11	755.1827	757.1980	C_36_H_36_O_18_	Chrysin-C-(O-galloyl-monoacetyldeoxyhexosyl) hexoside 1
8.27	593.1563 *	549.1608	C_26_H_28_O_13_	Chrysin-O-hexosyl pentoside
8.28	959.2485	961.2614	C_44_H_48_O_24_	Chrysin-C-(O-diacetyldeoxyhexosyl) hexoside-O-galloyl hexoside
8.66	921.2297 **	809.2504	C_37_H_44_O_20_	Chrysin-C-(O-diacetyldeoxyhexosyl) hexoside-O-hexoside 2
8.69	973.2639	975.2770	C_45_H_50_O_24_	Chrysin-C-(O-diacetyldeoxyhexosyl) hexoside-O-methylgalloyl hexoside
8.76	943.2523	945.2665	C_44_H_48_O_23_	Chrysin-C-(O-diacetyldeoxyhexosyl) hexoside-O-galloyl deoxyhexoside
8.81	645.1831	647.1976	C_31_H_34_O_15_	Chrysin-C-(O-diacetyldeoxyhexosyl) hexoside 1
8.84	755.1815	757.1980	C_36_H_36_O_18_	Chrysin-C-(O-galloyl-monoacetyldeoxyhexosyl) hexoside 2
8.91	741.2047	743.2187	C_36_H_38_O_17_	Chrysin-C-(O-syringyl-deoxyhexosyl) hexoside
9.05	645.1830	647.1976	C_31_H_34_O_15_	Chrysin-C-(O-diacetyldeoxyhexosyl) hexoside 2
9.06	645.4419	647.1976	C_31_H_34_O_15_	Chrysin-C-(O-diacetyldeoxyhexosyl) hexoside 3
9.27	1001.2589	1003.2719	C_46_H_50_O_25_	Chrysin-C-(O-triacetyldeoxyhexosyl) hexoside-O-galloyl hexoside
9.47	783.2140	785.2293	C_38_H_40_O_18_	Chrysin-C-(O-syringyl-monoacetyldeoxyhexosyl) hexoside
9.55	849.2453	851.2610	C_39_H_46_O_21_	Chrysin-C-(O-triacetyldeoxyhexosyl) hexoside-O-hexoside
9.57	769.1979	771.2136	C_37_H_38_O_18_	Chrysin-C-(O-methylgalloyl-monoacetyldeoxyhexosyl) hexoside
9.71	985.2633	987.2829	C_46_H_50_O_24_	Chrysin-C-(O-triacetyldeoxyhexosyl) hexoside-O-galloyl deoxyhexoside
9.72	1015.2743	1017.2876	C_47_H_52_O_25_	Chrysin-C-(O-triacetyldeoxyhexosyl) hexoside-O-methylgalloyl hexoside
9.90	1011.2792	1013.2927	C_48_H_52_O_24_	Chrysin-C-(O-triacetyldeoxyhexosyl) hexoside-O-caffeoyl hexoside

* formic acid adduct; ** diformic acid and sodium adduct; rt: retention time (min).

**Table 2:** Chrysin content in *Oncidium sotoanum* organs. FW = fresh weight.

	Flowers	Leaves	Pseudobulbs
Chrysin equivalents (mg g^−1^ FW)	10.5 ± 2.1	0.6 ± 0.2	0.5 ± 0.3

**Table 3:** Chrysin content of different plant materials. DW = dry weight; FW = fresh weight.

Species	Material	Chrysin Content	Ref.
*Hyphaene thebaica*	Male flowers	83 µg/g DW	[47]
*Juglans regia*	Diaphragma juglandis (from walnuts)	3.18 µg/g DW	[48]
*Cytisus villosus* Pourr *	Aerial parts	4 µg/g DW	[37]
*Momordica charantia*	Ripe fruit pulp	11 µg/g DW	[49]
	Ripe fruit peel	39 µg/g DW	
	Ripe fruit seed	24 µg/g DW	
	Unripe (whole fruit)	9 µg/g DW	
*Bulbophyllum odoratissimum*	Air-dried whole plant	27 µg/g DW	[50]
*Cypripedium macranthos* var. *rebunense*	In vitro regenerated plantlets	0.5 mg/g FW	[51]
*Desmos cochinchinensis*	Shade-dried leaves	14 µg/g DW	[52]

* For chrysin-7-*O*-β-d-glucopyranoside: 15.9 mg/g DW [53].

**Table 4:** Extract dilutions tested in FRAP and DPPH assays.

Organ	FRAP	DPPH
Leaves	1:10	1:5
Flowers	1:10	1:2
Roots	1:10	crude extract
Pseudobulbs	1:10	crude extract

## Data Availability

Not applicable.

## Supplementary Materials

The following supporting information can be downloaded at: https://www.mdpi.com/article/10.3390/plants12030655/s1, Supplementary File S1: LC-HRMS datamatrix with putatively identified metabolites.
